# The Effect of the Imacoco Care Psychoeducation Website on Improving Psychological Distress Among Workers During the COVID-19 Pandemic: Randomized Controlled Trial

**DOI:** 10.2196/33883

**Published:** 2022-03-10

**Authors:** Kotaro Imamura, Natsu Sasaki, Yuki Sekiya, Kazuhiro Watanabe, Asuka Sakuraya, Yutaka Matsuyama, Daisuke Nishi, Norito Kawakami

**Affiliations:** 1 Department of Mental Health Graduate School of Medicine The University of Tokyo Tokyo Japan; 2 Department of Public Health Kitasato University School of Medicine Sagamihara Japan; 3 Division of Public Health Department of Hygiene and Public Health, School of Medicine Tokyo Women's Medical University Tokyo Japan; 4 Department of Biostatistics Graduate School of Medicine The University of Tokyo Tokyo Japan; 5 Department of Public Mental Health Research National Institute of Mental Health National Center of Neurology and Psychiatry Tokyo Japan

**Keywords:** COVID-19, education, internet-based intervention, occupational groups, psychological distress, mental health, digital health, health intervention, psychoeducation

## Abstract

**Background:**

The prolonged COVID-19 pandemic has affected mental health among workers. Psychoeducational intervention via a website could be effective for primary prevention of mental illness among workers in the current COVID-19 pandemic.

**Objective:**

The aim of this randomized controlled trial is to examine the effect of a newly developed online psychoeducational website named Imacoco Care on reducing psychological distress and fear about COVID-19 infection among workers.

**Methods:**

Participants in the study were recruited from registered members of a web survey company in Japan. Participants who fulfilled the eligibility criteria were randomly allocated to the intervention or control group. Participants in the intervention group were invited to access the Imacoco Care program within a month after the baseline survey. The Kessler Psychological Distress Scale (K6) and the Fear of COVID-19 Scale (FCV-19S) scores were obtained at baseline and at 1- and 3-month follow-ups.

**Results:**

A total of 1200 workers were randomly allocated to the intervention and control groups (n=600 [50%] per group). The Imacoco Care intervention group showed a significant favorable effect on K6 scores (*P*=.03) with a small effect size (ES; Cohen *d*=–0.14) and an adverse effect on FCV-19S scores (*P*=.01) with a small ES (Cohen *d*=0.16) at 3-month follow-up. In the per protocol analysis (including only participants who had read the Imacoco Care content at least 1 time), the Imacoco Care intervention group also showed a significant favorable effect on reducing K6 scores (*P*=.03), while an adverse effect on FCV-19S scores was not significant (*P*=.06) in the intervention group at 3-month follow-up.

**Conclusions:**

A web-based psychoeducation approach may be effective for improving psychological distress among workers; however, it may be important not only to distribute information but also to encourage active engagement with the content of the program to prevent adverse effects of psychoeducational intervention.

**Trial Registration:**

University Hospital Medical Information Network Clinical Trials Registry (UMIN-CTR) UMIN000042556; https://upload.umin.ac.jp/cgi-open-bin/ctr_e/ctr_view.cgi?recptno=R000048548

## Introduction

### Background

The prolonged COVID-19 pandemic has affected the mental health not only of essential health care workers [1-5] but also of other workers (ie, non-health-care) [6,7]. Previous studies have reported a high prevalence of depression (34.6%), anxiety (42.3%), and psychological distress (65.1%) among workers [6,7], as well as a decline in the health-related quality of life [8] during the COVID-19 pandemic. Providing effective primary prevention stress management interventions is essential for protecting and promoting the mental health of workers during the prolonged COVID-19 pandemic [9].

Psychoeducational intervention is 1 of the effective treatments for primary prevention of mental health problems in the working population. A recent systematic review aiming to identify and summarize interventions for dealing with mental health issues of health care workers during infectious disease outbreaks, including COVID-19, reported that emotional and psychological intervention, including psychoeducation, is an important approach [10], especially since the 2000s, online-delivered psychoeducational interventions have received much attention. A recent meta-analysis reported that online psychological and psychoeducational interventions, which are mainly based on the principle of cognitive behavioral therapy (CBT), have a significant small effect (pooled standardized mean difference [SMD] –0.26) on reducing depressive symptoms in nondepressed and varied populations, with a moderate quality of evidence [11]. One of the effective online formats for delivering psychoeducational information is a website. Some previous studies before the COVID-19 pandemic have reported that psychoeducation websites significantly improved depressive symptoms among community residents with elevated depressive symptoms [12] and workers who visited a mental health specialist in the past month at baseline [13]. Psychoeducational intervention via a website could also be effective for primary prevention among workers in the current COVID-19 pandemic.

Although online/website-based psychoeducational intervention may be promising for improving the mental health of workers in the COVID-19 pandemic, only a few randomized controlled trials (RCTs) have been conducted during the COVID-19 pandemic. Regarding studies targeting community residents, 2 RCTs applying CBT-based intervention among people with elevated mental symptoms showed favorable effects on mental health outcomes [14,15], while another RCT applying low-intensity online mindfulness-based intervention in the general population could not find a significant effect on any outcomes [16]. In the working population, only 1 study focusing on health care workers who had provided direct face-to-face health care to patients with a diagnosis of infection with COVID-19 was conducted during the COVID-19 pandemic [17]. This RCT reported that a self-guided CBT and mindfulness-based mobile health intervention (the PsyCovidApp) significantly improved overall mental symptoms (total score of depression, anxiety, and stress) among health care workers who used psychotropic medications (SMD –0.29) or received psychotherapy (SMD –0.25) at baseline, while there was no significant intervention effect among the whole sample [17]. Developing an effective online psychoeducational intervention and examining its effect on promoting mental health are needed for workers during the COVID-19 pandemic. Although demonstrating the efficacy of such a cost-effective psychoeducation website would be beneficial, there is no other RCT aiming to examine the effect of a psychoeducational website on improving mental health problems in the working population in the current COVID-19 pandemic. Therefore, a further RCT is needed to examine the effect of psychoeducational intervention in a sample of workers.

### Objective

The aims of this study were to examine the effect of a newly developed self-guided, low-intensity, and easy-to-disseminate psychoeducational website named Imacoco Care on improving mental health (ie, reducing psychological distress and fear about COVID-19 infection) among workers, using an RCT design.

The hypotheses in this study were as follows: (1) Imacoco Care would reduce psychological distress as the primary outcome among workers, and (2) Imacoco Care would reduce fear about COVID-19 as the secondary outcome among workers.

## Methods

### Trial Design

This study was an RCT. The allocation ratio of the intervention group to the control group was 1:1. The study protocol was registered at the University Hospital Medical Information Network Clinical Trials Registry (UMIN000042556). This paper was reported according to the Consolidated Standards of Reporting Trials (CONSORT) guidelines [18,19].

### Ethical Considerations

The Research Ethics Review Board of the Graduate School of Medicine and Faculty of Medicine, University of Tokyo, approved the study procedures (number 3083-6).

### Participants

Participants in this study were recruited from registered members of a web survey company in Japan (Rakuten Insight, Inc [20]). Over 2 million members are registered in this company. Of those, 10,000 adult workers who were potentially eligible for the study were randomly selected. These candidates received a URL of a webpage that included detailed information about the study. The aims and procedures of the study were fully explained on the webpage. Participants were asked to click the Agree button to show their consent to participate in the study; then they proceeded to the baseline questionnaire page. Written consent was not required by the National Ethical Guidelines for Epidemiologic Research, Japan; the Research Ethics Review Board of the Graduate School of Medicine and Faculty of Medicine, University of Tokyo, approved this procedure for obtaining participants’ consent.

#### Eligibility Criteria of This Study

The inclusion criteria of this study were (1) adults (over 20 years old) and (2) full-time employees. The exclusion criteria were (1) having 15 sick leave days or more during the past 3 months, (2) having consultations with mental health professionals during the past month, and (3) having participated in this intervention program in the past.

### Intervention Program: Imacoco Care

Imacoco Care is a newly developed self-guided psychoeducation website-based program for providing information about coping with stress or problems under the conditions of the COVID-19 pandemic [21]. Imacoco Care provides information for managing stress and maintaining mental health among people staying at home under the state of emergency. The content is mainly composed of text, illustrations, video, and audio narration. There are about 30 pages in total, with around 1000 Japanese characters (500 words in English) per page. All content is explained in plain language, easy to understand for most workers, and does not presume prior knowledge of stress management.

Imacoco Care provides information about evidence-based psychological interventions for psychological distress (see Table 1), and the website can be accessed anywhere the internet is available. All of the Imacoco Care content was developed by the authors. Recent studies focusing on mental health among workers in the COVID-19 pandemic have reported that psychological/physical interventions, such as mindfulness, CBT-based psychoeducation, and physical activity, could improve mental health problems during the COVID-19 pandemic [14,22,23]. Details of the content included on the Imacoco Care website are listed in Table 1.

**Table 1:** Overview of the content of the Imacoco Care website.

Modules	Content
Mindfulness	This module provides information about the benefits of mindfulness and how to engage in it with easy-to-understand audio and video.
Behavioral activation (BA)	This module introduces how to relate behaviors/actions to feelings, how to maintain mental energy, and how to perform activities that lead to good feelings.
Physical activity	This module provides knowledge and tips for leading an active life while people are asked to stay at home, being properly aware of the spread of the new coronavirus.
Sleep education	This module provides information about how to regulate the sleep rhythm without using medication.
Tips for working from home	This module provides tips to make telecommuting (working from home) a little more comfortable.
Coping with the stress about COVID-19	This module provides stress management tips that can be practiced in daily life.

#### Mindfulness

An operational definition of mindfulness is “the awareness that emerges through paying attention on purpose, in the present moment, and nonjudgmentally to the unfolding of experience moment by moment” [24]. A previous meta-analysis reported that mindfulness meditation programs yielded moderate evidence of improved anxiety (effect size [ES]=0.38 at 8 weeks and 0.22 at 3-6 months) and depression (ES=0.30 at 8 weeks and 0.23 at 3-6 months) [25]. Imacoco Care provides information about the benefits of mindfulness and how to engage in it in the following lessons, with easy-to-understand audio and video: (1) For When You Feel Uncomfortable—an Introduction to Mindfulness; (2) The Body Relates to Our Feelings—Using mindfulness to Pay Attention to the Sensations of the Body; (3) Mindfulness by Narrating Like a Live Broadcast; (4) Mindful Eating; and (5) Self-Compassion Exercises to Calm Painful Feelings [26].

#### Behavioral Activation

Behavioral activation (BA), one of the most readily applied techniques in CBT, is a process to increase pleasurable and rewarding activities using behavioral strategies, such as activity scheduling [27]. A previous meta-analysis showed that CBT including BA could significantly reduce the risk of the onset of major depression (incidence rate ratio=0.62) [28]. Imacoco Care introduces how to relate behaviors/actions to feelings, how to maintain mental energy, and which activities lead to good feelings in the following lessons: (1) Tips for Keeping Your Mind Energized During Difficult Daily Life, (2) The Relationship Between What You Do or Don’t Do and Your Feelings, (3) Reflecting on Your Life Patterns, (4) Think of a “Safe Activity” That Doesn’t Fit Into the “Three Cs” (Crowded Places, Close Contact, Confined and Enclosed Spaces), (5) Let’s Make Your Action Plan!, and (6) Reflections: Tips for Acting to Keep Energy in Your Life.

#### Physical Activity

Physical activity is defined as any bodily movement produced by skeletal muscles that requires energy expenditure, which encompasses moderate-vigorous exercise and lower-intensity walking [29]. A previous meta-analysis showed that physical activity reduced depression by a medium effect (SMD –0.50) and anxiety by a small effect (SMD –0.38) [30]. Doing physical activity is also recommended during the COVID-19 outbreak [31]. Imacoco Care provides knowledge and tips for leading an active life while people are asked to stay at home and being properly aware of the spread of the new coronavirus, in the following lessons: (1) Physical Activity for Mental Health—Introduction; 2) Let’s Find a Physical Activity That Suits You—for Beginners; and (3) Let’s Find a Physical Activity That Suits You—for Advanced Learners.

#### Sleep Education

Some studies have shown that sleep problems or insomnia were detected in around 35% of participants (ie, community residents or health care workers) during the COVID-19 pandemic [32,33]. CBT for insomnia (CBT-I) is one of the effective treatments for improving sleep problems, and it is recommended as the first-choice treatment for insomnia in the COVID-19 outbreak [34]. Imacoco Care provides information about how to regulate the sleep rhythm without using medication in the following lessons: (1) Psychological Advice for Those Whose Sleep Cycle Has Become Worse and (2) Life Tips to Try When You Can’t Sleep.

#### Tips for Working From Home

Previous literature reviews have reported that the effects of working at home are inconsistent and might depend on various factors, such as demands of the home environment, the level of organizational support, and social connections external to work, among others [35]. This module provides some tips to make telecommuting (working from home) a little more comfortable, such as ergonomic tips when teleworking, keeping the time of the lunch break, getting fresh air, stretching and exercising, and keys for effective communication [36,37].

#### Coping With the Stress About COVID-19

This module provides stress management tips that can be practiced in daily life, including (1) prioritize personal hygiene (preventive measures), (2) stay connected with others, (3) keep a healthy lifestyle, (4) use psychological coping strategies (eg, mindfulness), (5) limit media consumption, and (6) prevent stigma and discrimination [36,38].

### Intervention Group

Immediately after the baseline survey, participants in the intervention group were invited to access Imacoco Care and requested to view Imacoco Care within 1 month after the baseline survey. During this period, the participants received a reminder email twice (1 week after the baseline survey and 1 week before the 1-month follow-up survey).

### Control Group

Participants in the control group were only asked to complete the baseline and follow-up surveys. They were not invited to the Imacoco Care website, but they were not restricted from accessing any other information or service as treatment as usual (TAU).

### Outcome Measures

All outcomes were measured using a web-based self-report questionnaire at baseline, 1-month follow-up, and 3-month follow-up.

### Primary Outcomes

#### Psychological Distress

The Kessler Psychological Distress Scale (K6) consists of 6 items assessing the frequency with which participants experience symptoms of psychological distress during the past 30 days [39,40]. The response options range from 0 (none of the time) to 4 (all the time). The internal reliability and validity found in previous studies are acceptable [39].

#### Fear of COVID-19 Infection

The Fear of COVID-19 Scale (FCV-19S) consists of 7 items assessing the fear of COVID-19 [41,42]. The response options range from 1 (strongly disagree) to 5 (strongly agree), with total scores ranging from 7 to 35. The higher the score, the greater the fear of COVID-19. The internal reliability and validity found in previous studies are acceptable [43].

### Process Evaluation

#### Implementation Aspects of the Intervention Program, the Number of Views, and Progress in Learning About Imacoco Care

The Implementation Outcome Scale for Digital Mental Health (iOSDMH) for users was used to assess the implementation aspects of Imacoco Care [44]. The iOSDMH for users consists of 19 items assessing acceptability (3 items), appropriateness (4 items), feasibility (6 items), satisfaction (1 item), and harm (5 items). The response options range from 1 (disagree) to 4 (agree). The number of views was evaluated by using 1 item: “How many times did you view Imacoco Care during the past month?” There were 4 response options (never, once, twice, and 3/4 times or more). This scale was originally developed and has not yet been validated. To assess progress in learning about each of the 6 modules of Imacoco Care, participants in the intervention group were also asked, “Have you read and tried each content of Imacoco Care?” There were 3 response options (have not read, only read, and read and tried). This scale was also originally developed and has not yet been validated. These questions were asked of only participants in the intervention group in the 1-month follow-up survey.

#### Contamination of Information

To evaluate the degree of contamination of information, the control group participants were asked the following question in 1- and 3-month follow-up surveys: “During the past, have you ever viewed a website named Imacoco Care?” There were 4 response options (none, once, twice, and 3/4 times or more). This scale was originally developed and has not yet been validated.

### Demographic Characteristics

Demographic data, such as age, gender, marital status (never married, married, divorced, or bereaved), education (high school or lower, some college, university, graduate school or higher), occupational status (manager, professional, clerical, production, sales, others), working style (working from home only, both working from home and at the office, working at the office only), and chronic disease (yes/no) were collected in the baseline survey.

### Sample Size Calculation

The required sample size was calculated for psychological distress assessed by K6. A previous meta-analysis of an unguided eHealth intervention on improving workers' mental health in the workplace yielded an ES of 0.22 [45]. To detect a small ES (ie, 0.2) or more at an α error rate of .05 and a β error rate of .10, the estimated sample size was 527 participants in each group. The statistical power was calculated using the G*Power 3.1 program [46,47].

### Randomization

Participants who fulfilled the eligibility criteria were randomly allocated to 1 of 2 arms (intervention or control group). Stratified permuted-block randomization was conducted. The block sizes of this study were fixed to 2. Participants were stratified into 2 strata according to the K6 score (<4 or ≥5) in the baseline survey [48]. A stratified permuted block random table was generated by an independent biostatistician. Enrollment was conducted by a clinical research coordinator, and assignment was conducted by an independent research assistant. The stratified permuted-block random table was password-protected and blinded to the researcher. Only the research assistant could access it during the work of random allocation.

### Statistical Methods

For the main analysis, a mixed model for repeated measures (MMRM) analysis of variance model was conducted using a group (intervention and control) × time (baseline, 1-month follow-up, and 3-month follow-up) interaction as an indicator of the intervention effect. Missing values at follow-up surveys were imputed applying the maximum likelihood estimation using the mixed procedure. An intention-to-treat (ITT) principle was applied.

The ES was estimated in 2 ways. First, we estimated a regression coefficient for a group (intervention vs control group) × time (baseline and 2 follow-ups) interaction using the mixed procedure, which was converted to an ES by dividing by a pooled SD at baseline and at follow-ups. Second, we calculated the Cohen *d* values [49] among participants who completed surveys at baseline and at each follow-up. The level of statistical significance for all analyses in this study was set at .05 (two-tailed), and 95% CIs were calculated. All statistical analyses were conducted using SPSS Statistics V.26.0 (IBM Corp).

#### Per Protocol Analysis

For the per protocol analysis, the same MMRM analysis was conducted using only participants who had viewed the Imacoco Care website at least once in the intervention group and all participants in the control group.

## Results

### Participant Recruitment

**Figure 1:**
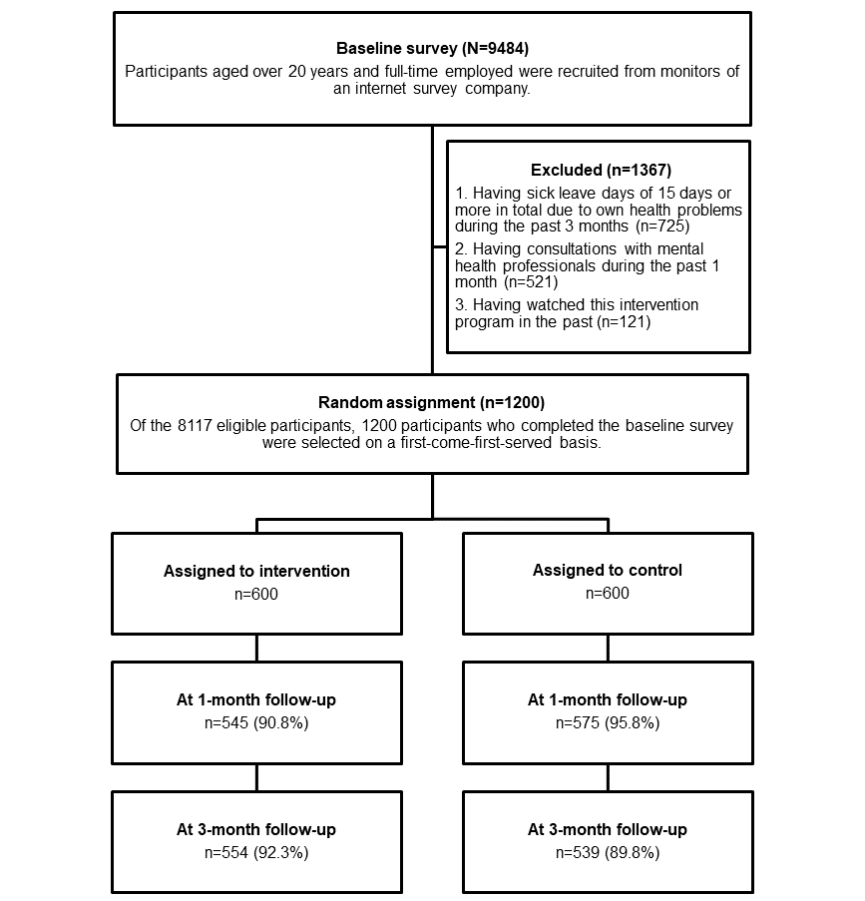
Participant flow.

The participant flowchart is shown in Figure 1. Recruitment and the baseline survey were conducted in December 2020. Follow-up surveys in both groups were conducted 1 month (January 2021) and 3 months (March 2021) after the baseline survey. Participants who had fulfilled the inclusion criteria (ie, aged over 20 years and full-time employed) were recruited from monitors of an internet survey company (N=9484). Of those, 1367 (14.41%) were excluded according to the exclusion criteria. Of the 8117 eligible participants, 1200 (14.75%) who completed the baseline survey were selected on a first-come-first-served basis. Participants were randomly allocated to an intervention or a control group, with 600 (50%) participants in each.

### Baseline Characteristics

Demographic characteristics are presented in Table 2. In the whole sample, most participants were male, were married, received university or higher education, and did not report having chronic diseases. Only less than 30% were using telecommuting. Between the 2 groups, demographic characteristics of participants were similar. About 192 (32%) participants in each group had psychological distress (ie, scored 5 or more on K6).

**Table 2:** Baseline characteristics of participants in the intervention and control groups.

Variables		Intervention (N=600)	Control (N=600)
Age (years), mean (SD)		46.4 (10.3)	46.7 (10.0)
**Gender, n (%)**			
	Male	441 (73.5)	442 (73.7)
	Female	159 (26.5)	158 (26.3)
**Marital status, n (%)**			
	Never married	190 (31.7)	190 (31.7)
	Married	362 (60.3)	369 (61.5)
	Divorced/bereaved	48 (4.0)	41 (6.8)
**Education, n (%)**			
	High school	117 (19.5)	125 (20.8)
	Some college	105 (17.5)	108 (18.0)
	University	312 (52.0)	308 (51.3)
	Graduate school	66 (11.0)	59 (9.8)
**Occupation, n (%)**			
	Manager	122 (20.3)	135 (22.5)
	Professional	186 (31.0)	164 (27.3)
	Clerical	157 (26.2)	140 (23.3)
	Production	57 (9.5)	66 (11.0)
	Sales	61 (10.2)	64 (10.7)
	Others	17 (2.8)	31 (5.2)
**Working style, n (%)**			
	Working from home only	27 (4.5)	28 (4.7)
	Both working from home and at the office	133 (22.2)	136 (22.7)
	Working at the office only	440 (73.3)	436 (72.7)
**Chronic disease, n (%)**			
	Yes	116 (19.3)	100 (16.7)
	No	484 (80.7)	500 (83.3)
	5 or more on K6 score	192 (32.0)	193 (32.2)

### Effect of the Imacoco Care Program on Psychological Distress

The average K6 and FCV-19S scores in each survey are shown in Table 3. The K6 score increased at 1-month follow-up in both groups and decreased at 3-month follow-up only in the intervention group. The Imacoco Care intervention group showed a significant ES in reducing the K6 score at 3-month follow-up compared to the control group (Cohen *d*=–0.14, 95% CI –0.26 to –0.02). The FCV-19S score also increased at 1-month follow-up and decreased at 3-month follow-up in both groups. The Imacoco Care intervention group showed a significant adverse ES in increasing the FCV-19S score at 3-month follow-up compared to the control group (Cohen *d*=0.16, 95% CI 0.04-0.28).

Table 4 shows the estimated effects of the Imacoco Care intervention on K6 and FCV-19S scores on the basis of MMRM analyses. In ITT analyses, the Imacoco Care intervention showed a significant favorable effect on reducing the K6 score (*P*=.03) and an adverse effect on the FCV-19S score (*P*=.01) in the intervention group at 3-month follow-up. In the per protocol analyses, the Imacoco Care intervention also showed a significant favorable effect on reducing the K6 score (*P*=.03), while the adverse effect on the FCV-19S score was not significant (*P*=.06) in the intervention group at 3-month follow-up.

**Table 3:** Means (SDs) of outcome variables at baseline, 1-month follow-up, and 3-month follow-up in the intervention and control groups.

Outcome variables			Intervention			Control			ES^a^
		n (%)		Mean (SD)	n (%)		Mean (SD)	Cohen *d* (95% CI)	
**Psychological distress**									
	Baseline	600 (100)		3.8 (4.7)	600		3.7 (4.4)	N/A^b^	
	1-month follow-up	545 (90.8)		4.2 (5.0)	575		4.4 (5.0)	–0.07 (–0.19 to 0.04)	
	3-month follow-up	554 (92.3)		3.8 (4.9)	539		4.3 (5.2)	–0.14 (–0.26 to –0.02)	
**Fear about COVID-19**									
	Baseline	600 (100)		14.6 (5.6)	600		15.4 (5.8)	N/A	
	1-month follow-up	545 (90.8)		15.9 (5.8)	575		16.3 (5.9)	0.10 (–0.02 to 0.22)	
	3-month follow-up	554 (92.3)		14.8 (5.8)	539		14.8 (6.0)	0.16 (0.04-0.28)	

^a^ES: effect size. Cohen *d* values were calculated among participants who answered each follow-up survey.

^b^N/A: not applicable.

**Table 4:** Effects of the Imacoco Care on outcomes.

Outcome variables			ITT^a^						Per protocol^b^				
		Effect (ES^c^)		95% CI	SE	*t* (*df*)	*P* value	Effect (ES)		95% CI	SE	*t* (*df*)	*P* value
**Psychological distress**													
	1 month	–0.30 (–0.08)		–0.76 to 0.17	0.24	–1.26 (1807.72)	.21	–0.39 (–0.10)		–0.98 to 0.20	0.30	–1.28 (1333.94)	.20
	3 months	–0.53 (–0.13)		–1.01 to –0.06	0.24	–2.21 (1157.11)	.03	–0.67 (–0.17)		–1.30 to –0.05	0.32	–2.12 (797.91)	.03
**Fear about COVID-19**													
	1 month	0.46 (0.10)		–0.10 to 1.03	0.29	1.60 (1847.74)	.11	0.60 (0.13)		–0.13 to 1.33	0.37	1.61 (1328.98)	.11
	3 months	0.83 (0.16)		0.23-1.42	0.30	2.74 (1186.92)	.01	0.74 (0.15)		–0.03 to 1.52	0.39	1.89 (822.80)	.06

^a^ITT: intention to treat.

^b^Participants who saw the Imacoco Care website at least 1 time in the intervention group (n=235 [39.2%]) were included.

^c^ES: effect size, calculated by dividing the estimated effect by a pooled SD at baseline and at follow-ups.

### Process Evaluation

Among participants (n=545, 90.8%) in the intervention group at the 1-month follow-up, 52 (9.5%) viewed the Imacoco Care website 4 times or more, 96 (17.6%) viewed it 2 or 3 times, 87 (16.0%) viewed it once, and 310 (56.9%) never viewed the Imacoco Care website.

Among participants (n=235, 39.2%) who viewed the Imacoco Care website at least once, 13 (5.5%) read all the content, 82 (34.9%) read most of the content, 73 (31.1%) read some of the content, 66 (28.1%) read a little of the content, and 1 (0.4%) read little of the content. In addition, 174 (74.0%) read or tried mindfulness techniques, 159 (67.7%) read or tried BA, 170 (72.3%) read or tried physical activity, 195 (83.0%) read or tried sleep education, 121 (51.4%) read or tried the tips for working from home, and 165 (70.2%) read or tried techniques for coping with stress about COVID-19.

Regarding the result of implementation outcomes assessed by the iOSDMH, around 105-174 (44.7%-74.0%) of the 235 participants answered “agree” or “relatively agree” on each item of acceptability (n=105-158, 44.7%-67.2%), appropriateness (n=115-164, 48.9%-69.8%), and feasibility (n=135-174, 57.4%-74.0%). The highest proportions of positive responses in each of the 3 aspects were “Advantages outweigh the disadvantages for keeping my mental health” (n=158, 67.2%) in acceptability, “Appropriate (from your perspective, it is the right thing to do)” (n=164, 69.8%) in appropriateness, and “Lower physical effort” (n=174, 74.0%) in feasibility, respectively. Regarding overall satisfaction with Imacoco Care, 135 (57.4%) answered “agree” or “relatively agree.” Regarding harms, around 47 (20%) or fewer participants answered “agree” or “relatively agree” on each item, except for “Time-consuming” (n=63, 26.8%). The details of the number of participants for each item are shown in Multimedia Appendix 1.

Regarding the contamination of information, 9 (1.5%) of 600 participants in the control group had viewed the Imacoco Care website at least once at 1-month follow-up and 17 (2.83%) had viewed the Imacoco Care website at 3-month follow-up.

## Discussion

### Principal Findings

This large-scale RCT showed that the newly developed psychoeducational website during the COVID-19 pandemic, named Imacoco Care, significantly improved psychological distress at 3-month follow-up among workers in Japan, with a small ES. In concordance with previous studies conducted before the COVID-19 pandemic, a web-based psychoeducation approach including information about evidence-based psychological interventions may be effective for improving psychological distress (ie, depressive and anxiety symptoms) among workers in the current COVID-19 pandemic. The Imacoco Care website may promote mental health among workers, which mainly consisted of male, married, university graduates without chronic disease or current mental health problems, during the COVID-19 pandemic.

### Comparison With Prior Work

To our knowledge, this study is the first to examine whether a psychoeducational website can improve psychological distress symptoms among workers during the COVID-19 pandemic. A significant intervention effect of the Imacoco Care intervention was found on psychological distress at 3-month follow-up, while the ES was small (Cohen *d*=–0.16) compared to previous studies of participants with elevated psychological distress or worry about COVID-19 (around 0.7) [14,15]. One of the possible reasons for the small ES in this RCT may be the lower intensity of the intervention. A previous RCT provided 5 modules with a few tasks to practice. Participants were encouraged to report on their progress in digital worksheets in the online platform [14]. The other pilot RCT provided 7 selected modules out of 16 possible modules, with a therapist’s (ie, clinical psychologist) support. Each participant had a therapist who provided online support and feedback on the work with the modules and exercises and motivated the participant to continue to work with the treatment [15]. The Imacoco Care program only provided information via the website, and participants received a reminder email only twice. This may have led to a small ES; nevertheless, the Imacoco Care program still has merit because of its greater accessibility and lower cost. The other possible reason may be the difference in the baseline characteristics of the participants. The study excluded workers with current mental health problems (ie, having 15 sick leave days or more during the past 3 months or consultations with mental health professionals during the past month). This may have led to an underestimation of the intervention effect (floor effect).

Regarding fear about COVID-19, Imacoco Care showed a significant adverse effect (Cohen *d*=0.16) at 3-month follow-up. This slight increase in the fear about COVID-19 may be due to increased awareness of stress caused by learning from the Imacoco Care program [13,50]. In contrast, the result from per protocol analysis that included only participants who had read the Imacoco Care website at least 1 time showed a nonsignificant adverse effect on the fear of COVID-19 at 3-month follow-up (Cohen *d*=0.15). An adverse effect on the fear about COVID-19 may have been more common among participants who never saw the Imacoco Care website during the intervention period. Both distributing the information and encouraging people to access and read the content may be necessary to prevent adverse effects of psychoeducational intervention. Users should be informed about the possible adverse effects of seeing the Imacoco Care website in advance to decide whether to take the program, balancing merits and demerits.

Process evaluation of the Imacoco Care program showed that the proportion of those who viewed the Imacoco Care website at least once was 235 (39.2%) of 600 participants. Of those, only 95 (40.4%) read all or most of the content on the Imacoco Care website. The low rates of reading the content may weaken the intervention effect. Regarding the results from per protocol analyses, the Imacoco Care program showed a greater effect for psychological distress (Cohen *d*=–0.17) among workers who had viewed the Imacoco Care program at least once. Increasing readers may be effective in improving the effect of Imacoco Care. From the implementation aspects, Imacoco Care showed moderate rates (around 105-174 [44.7%-74.0%]) of acceptability, appropriateness, and feasibility among participants who viewed the Imacoco Care website at least once. The features of the Imacoco Care program (explaining evidence-based psychological intervention in an easy-to-understand way by using plain language, illustrations, video, and audio narration) may contribute to these implementation outcomes. Further study is needed to develop an implementation strategy for increasing adoption of the Imacoco Care program among workers and examine its effect on implementation outcomes.

### Limitations

Several limitations of this study should be considered. First, all participants were recruited from registered members of a web survey company in Japan. Most of participants were male, married, and university graduates without chronic disease and psychological distress at the baseline survey. Therefore, generalization of the findings to the general working population may be limited. Second, website access logs by users were not collected. The actual proportion of access to each content area of Imacoco Care could not be analyzed. The effectiveness of each content area could not be examined, and the results from process evaluation may be biased. Third, all outcomes were measured by self-report, which may be affected by the perceptions of the participants or by situational factors at work. A further RCT and implementation study should be conducted to examine whether psychoeducational intervention via an online program is effective among workers with diverse characteristics, particularly in terms of education.

### Conclusion

An RCT was used to evaluate a new website-based psychoeducational program, named Imacoco Care, which was developed to address mental health issues during the COVID-19 pandemic. Results showed significantly reduced psychological distress at 3-month follow-up among workers in Japan, with a small ES. Thus, a web-based psychoeducation approach may be an effective intervention for reducing psychological distress in the working population. To enhance outcomes from this type of intervention and reduce any adverse effects, it may be important to not only distribute information about the program but also encourage workers to access and actively engage with the content of the program.

## Appendix

Multimedia Appendix 1 Supplemental table.

Multimedia Appendix 2 CONSORT-eHEALTH checklist (V 1.6.1).

## Abbreviations

BA: behavioral activation
CBT: cognitive behavioral therapy
ES: effect size
FCV-19S: Fear of COVID-19 Scale
iOSDMH: Implementation Outcome Scales for Digital Mental Health
ITT: intention to treat
K6: Kessler Psychological Distress Scale
MMRM: mixed model for repeated measures
RCT: randomized controlled trial
SMD: standardized mean difference
