# Supplementary material for: The Effect of the Imacoco Care Psychoeducation Website on Improving Psychological Distress Among Workers During the COVID-19 Pandemic: Randomized Controlled Trial
Source: JMIR Form Res. 2022 Mar 10;6(3):e33883. doi: 10.2196/33883 (PMC8949678; doi:10.2196/33883)
Supplement: Multimedia Appendix 1 [file formative_v6i3e33883_app1.docx]

Supplemental table 1 The number and proportions of responses of each option on iOSDMH (N=235).

| Short descriptions of the items | Disagree | Relatively disagree | Relatively agree | Agree |
| --- | --- | --- | --- | --- |
|  | n (%) | n (%) | n (%) | n (%) |
|  |  |  |  |  |
| **Acceptability** |  |  |  |  |
| Advantages outweigh the disadvantages for keeping my mental health. | 11 (4.7) | 66 (28.1) | 147 (62.6) | 11 (4.7) |
| Improves my social image. | 26 (11.1) | 104 (44.3) | 98 (41.7) | 7 (3.0) |
| Acceptable for me. | 14 (6.0) | 83 (35.3) | 124 (52.8) | 14 (6.0) |
| **Appropriateness** |  |  |  |  |
| Appropriate (from your perspective, it is the right thing to do). | 12 (5.1) | 59 (25.1) | 150 (63.8) | 14 (6.0) |
| Applicable to my health status. | 17 (7.2) | 100 (42.6) | 111 (47.2) | 7 (3.0) |
| Suitable for my social conditions. | 17 (7.2) | 96 (40.9) | 110 (46.8) | 12 (5.1) |
| Fits my living condition. | 15 (6.4) | 105 (44.7) | 109 (46.4) | 6 (2.6) |
| **Feasibility** |  |  |  |  |
| Easy to use. | 8 (3.4) | 92 (39.1) | 123 (52.3) | 12 (5.1) |
| Physical effort. ^a)^ | 47 (20.0) | 127 (54.0) | 57 (24.3) | 4 (1.7) |
| Total length is implementable. | 13 (5.5) | 87 (37.0) | 128 (54.5) | 7 (3.0) |
| Length of one content is implementable. | 12 (5.1) | 65 (27.7) | 150 (63.8) | 8 (3.4) |
| Frequency is implementable. | 9 (3.8) | 80 (34.0) | 141 (60.0) | 5 (2.1) |
| Easy to understand. | 11 (4.7) | 68 (28.9) | 142 (60.4) | 14 (6.0) |
| **Overall Satisfaction** |  |  |  |  |
| Satisfied with the program. | 11 (4.7) | 89 (37.9) | 124 (52.8) | 11 (4.7) |
| **Harms** ^a)^ |  |  |  |  |
| Physical symptoms. | 78 (33.2) | 115 (48.9) | 41 (17.4) | 1 (0.4) |
| Mental symptoms. | 90 (38.3) | 111 (47.2) | 33 (14.0) | 1 (0.4) |
| Induced dangerous experience regarding safety. | 92 (39.1) | 106 (45.1) | 29 (12.3) | 8 (3.4) |
| Time-consuming. | 64 (27.2) | 108 (46.0) | 59 (25.1) | 4 (1.7) |
| Excessive pressure on learning regularly. | 78 (33.2) | 105 (44.7) | 46 (19.6) | 6 (2.6) |

a) Negative item.
